# Leprosy neuropathy and demyelinating impairment: How should we interpret this neurophysiological pattern?

**DOI:** 10.1371/journal.pone.0343962

**Published:** 2026-04-08

**Authors:** Diogo Fernandes dos Santos, Iago Resende Carvalho, Isabella Sabião Borges, Pedro Henrique Sirotheau Corrêa Alves1, Fernanda de Oliveira Cirino, Douglas Eulálio Antunes, Raquel Campos Pereira, Marcus Vinicius Magno Gonçalves, Isabela Maria Bernardes Goulart

**Affiliations:** 1 National Reference Center for Sanitary Dermatology and Leprosy, Clinics’ Hospital, School of Medicine, Federal University of Uberlândia (UFU), Uberlândia, Minas Gerai, Brazil; 2 Postgraduate Program in Health Sciences, School of Medicine, Federal University of Uberlândia (UFU), Uberlândia, Minas Gerai, Brazil; 3 Professor of Neurology, School of Medicine, University of the Joinville (UNIVILLE), Joinville, South Carolina, Brazil; Lady Hardinge Medical College, INDIA

## Abstract

**Introduction/Aims:**

Leprosy neuropathy (LN) may cause demyelination that worsens during the leprosy reactions (LR). Type-1 LR (T1LR) occurs in patients with cell-mediated immune response against *M. leprae,* and Type-2 LR (T2LR) occurs in multibacillary cases. The patterns of nerve impairment need to be clarified, as both demyelination and axonal degeneration are commonly observed. This study aimed to describe how to interpret the demyelinating impairment in LN.

**Methods:**

Retrospective observational analysis of leprosy patients in a National Reference Center in Brazil between 2014–2023.

**Results:**

494 participants were included in this study. 3952 nerves were evaluated, with an average of 5.1 (±5.4) nerves affected per patient. 23.5% (116/494) of patients showed a demyelinating pattern defined by standard criteria, and 20.7% (24/116) presented exclusively demyelinating abnormalities without evidence of secondary axonal loss. 81% (94/116) presented conduction block, and 95.7% (111/116) temporal dispersion, with both conditions concomitant in 76.7% (89/116) of patients. 83.6% (97/116) demonstrated prolonged distal motor latency, and 99.1% (115/116) reduction in conduction velocity. 46.1% had T1LR and 17.9% T2LR. The comparison between patients with and without LR showed higher bacillary load, conduction block, and temporal dispersion in LR patients. 93.1% (108/116) of patients fulfilled neurophysiological criteria for chronic inflammatory demyelinating polyneuropathy (CIDP). Among them, 46.3% presented clinical criteria for atypical CIDP.

**Discussion:**

Leprosy is a spectral disease in which neural damage can manifest in different phenotypes. Demyelinating impairment is frequent and varies according to the clinical form and presence of LR. Although demyelinating impairment is common in the studied population, it does not reflect active disease. LN can also be misdiagnosed as other peripheral neuropathies, especially CIDP, in non-endemic areas.

## 1. Introduction

Leprosy is a chronic infectious disease caused by the *Mycobacterium leprae* (*M. leprae*), an obligatory intracellular bacillus. It is the main infectious etiology of peripheral neuropathy worldwide and still represents a serious public health problem in underdeveloped countries [1]. Despite being classically recognized for its cutaneous manifestations, leprosy must be treated as a primarily neural condition since the disabilities frequently observed in the disease are due to the presence of leprosy neuropathy [1,2].

Leprosy neuropathy (LN) is a spectral disease classified into different clinical forms based on the host’s immune response, histopathological features of the skin biopsy, and bacillary load. According to Ridley-Jopling’s classification, patients with a better cellular immune response to *M. leprae* are classified as tuberculoid (T). In contrast, patients with an anergic response are classified as lepromatous (L). Between these poles, there are borderline patients who present an intermediate immunological response, which can be classified as borderline-lepromatous (BL), borderline-tuberculoid (BT), and borderline-borderline (BB) [3,4].

Some patients may also present a form of the disease characterized by a chronic, progressive, asymmetric sensory-motor peripheral neuropathy, without cutaneous manifestations and with negative bacilloscopy of the slit skin smear. This form of presentation is recognized as primary neural leprosy (PNL) and represents a challenge in clinical practice, requiring differential diagnosis with other etiologies, especially chronic inflammatory neuropathies [5–7].

Peripheral nerve impairment is observed in all clinical forms of leprosy, but with varying presentations and severity. Clinically, it manifests as a non-length-dependent neuropathy with sensory predominance, configuring a pattern of asymmetric multiple mononeuropathy. Furthermore, neural thickening is expected, leading to LN being considered one of the main causes of hypertrophic neuropathy [8–11].

During the course of the disease, a significant proportion of patients experience acute inflammatory reactions that can occur before, during, or after treatment. These acute episodes are known as leprosy reactions (LR) and are differentiated into type 1 LR (T1LR) and type 2 LR (T2LR) [12–14].

T1LR is also called reverse reaction, which is marked by an abrupt increase in the cell-mediated immune response against *M. leprae* and occurs mainly in borderline patients (BT, BB, and BL). It is characterized by an acute worsening of preexisting skin and peripheral nerve lesions, or by the emergence of new ones. T2LR, also known as erythema nodosum leprosum (ENL), is a systemic inflammatory process that occurs in borderline and lepromatous patients and is associated with extravascular deposition of immune complexes. Patients present with erythematous and painful subcutaneous nodules accompanied by fever, hyporexia, and other systemic manifestations, such as orchitis, epididymitis, glomerulonephritis, myositis, arthralgia, iridocyclitis, hepatomegaly, and adenomegaly [14–16].

Considering the importance of recognizing neural damage in LN, electroneuromyography (ENMG) plays a prominent role in investigating and managing the disease. It allows stratification of severity, the definition of patterns of peripheral neural impairment, and early diagnosis in oligo/asymptomatic individuals [10,17]. The neurophysiological patterns found vary according to the clinical forms of the disease. However, in most cases, asymmetric sensory and motor axonal neuropathy is observed, associated with multiple demyelinating impairment [5,6,10,18–20].

This study aimed to describe the main electroneuromyographic findings compatible with neural impairment in leprosy patients (LP) and evaluate the frequency of this neurophysiological pattern in the LR.

## 2. Materials and methods

### 2.1. Ethical statement

This article has been approved by the Ethical Research Committee of the Federal University of Uberlandia (CAAE 45007721.7.0000.5152).

### 2.2. Type of study and subjects

**Fig 1:**
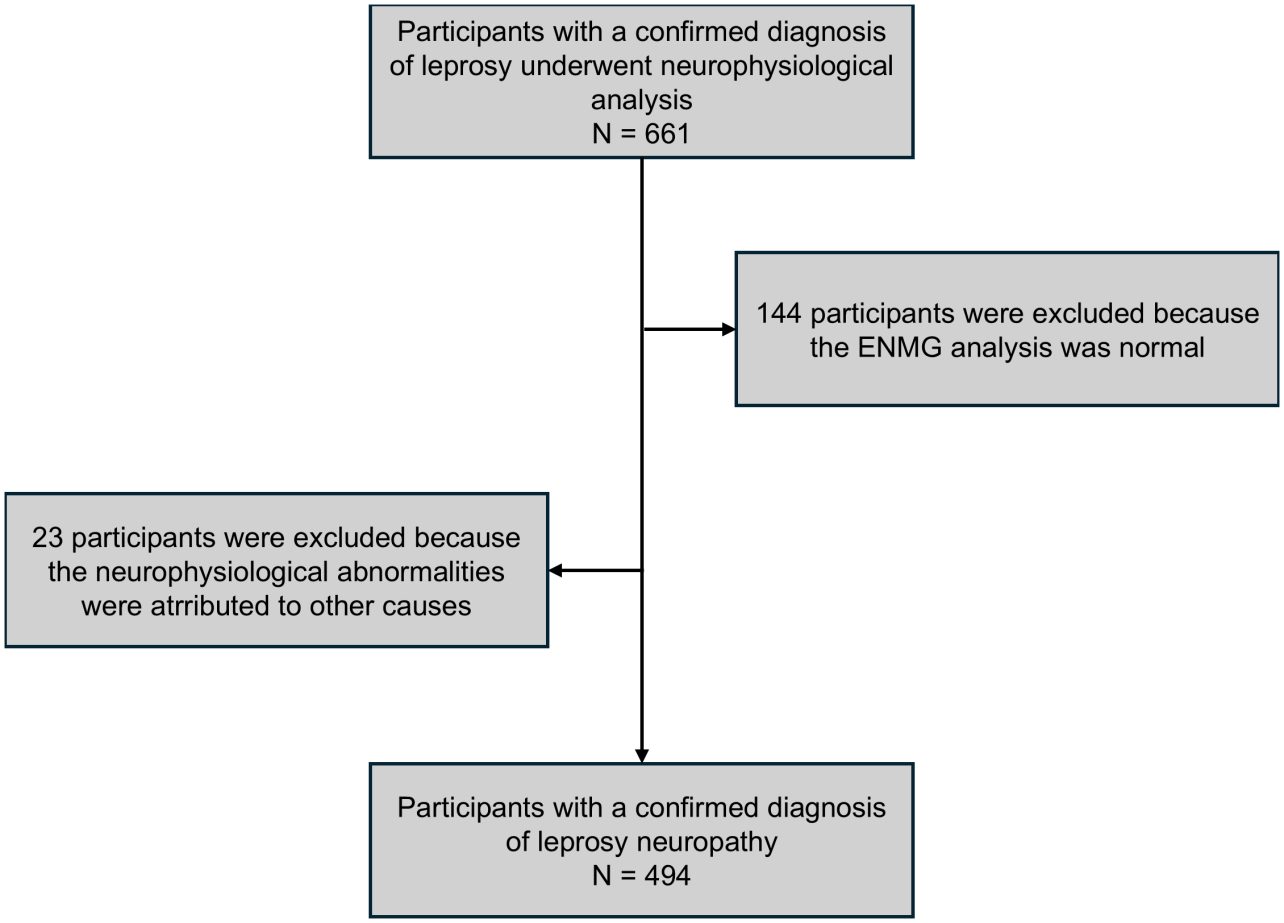
STROBE fluxogram showing the process of data selection.

This retrospective observational study comprises 494 leprosy patients who attended the outpatient clinic of a national reference center for leprosy in Brazil (Fig 1). The dataset covers the period between 2014 and 2023 and was accessed for research on January 25, 2024. As eligibility criteria, participants should have a confirmed leprosy diagnosis defined by the presence of at least one of the three cardinal signs: skin lesion with sensorial impairment, neural thickening associated with sensory or sensory-motor impairment, and presence of *M. leprae* confirmed by slit skin smear or skin biopsy bacilloscopy. All patients were also subjected to an extensive laboratory evaluation, utilizing serological and molecular tools to ensure accurate diagnosis, especially in cases where the slit skin smear and skin biopsy bacilloscopy results were negative. All patients with a confirmed diagnosis of leprosy who underwent electroneuromyographic evaluation were included. Patients who experienced a leprosy reaction during the course of the disease underwent ENMG between 4–8 weeks after the onset of the reaction. In patients without a leprosy reaction, ENMG was performed at the time of diagnosis. In cases where the patient experienced multiple reaction episodes, only the first electromyographic examination was evaluated.

We excluded patients who showed other possible etiologies of peripheral neuropathies, in addition to leprosy neuropathy (e.g., chronic alcoholism, diabetes mellitus, thyroid disease and other hormonal dysfunctions, malnutrition, hepatitis, HIV, and autoimmune diseases).

### 2.3. Epidemiological, clinical, and neurological assessment

Epidemiological data (sex, age), clinical data (clinical form, type of reaction, and previous treatment), neurological data (sensory and motor assessment), and disability grade were obtained from the patient's medical records. According to the Brazilian Ministry of Health protocol, all patients had undergone routine sensory and motor neurological evaluation [21]. This protocol includes a sensory and motor evaluation of the hands and feet, and assessment of neural thickening.

According to the WHO leprosy-related impairment classification, the level of functional disability is evaluated by assessing neural function integrity and the degree of physical disability through voluntary muscle testing and sensory evaluation of the hands and feet. Patients with no neural impairment are classified as disability grade zero (DG0), disability grade 1 (DG1) occurs when only sensorial impairment exists, and disability grade 2 (DG2), in which there are visible deficiencies, such as claws (claw of digits), bone resorption, muscle atrophy, contractures, and wounds [21].

### 2.3. Laboratory evaluation

Below, we describe all the laboratory methods routinely used in our service that facilitate early diagnosis of leprosy neuropathy and support an adequate differential diagnosis with other conditions.

#### 2.3.1. Bacilloscopy.

The bacillary load analysis was performed on slit skin smears from both ear lobes, elbows, and knees, and on skin and/or nerve biopsies, if clinical data were compatible.

#### 2.3.2. ELISA anti-PGL-I IgM serology.

ELISA detected serum anti-PGL-I IgM antibodies performed against the purified native PGL-I from the *M. leprae* cell wall. The reagent was obtained through BEI Resources, NIAID, NIH: Monoclonal Anti-*Mycobacterium leprae* PGL-I, Clone CS-48 (produced *in vitro*), NR-19370. The titration of anti-PGL-I antibodies was expressed as an ELISA index, defined as the ratio of the sample's bacillary load to the cutoff. Values above 1.0 were considered positive [22].

#### 2.3.3. DNA extraction and real-time quantitative PCR (qPCR).

DNA was extracted from dermal smear samples and biopsies (nerve and skin) and detected by quantitative real-time polymerase chain reaction (qPCR) primer/probe assay targeting the *M. leprae* species-specific genomic region (RLEP3) [14]. The reactions were performed on the ABI 7300 platform (Applied Biosystems), and the results were analyzed using the 7300 System SDS Software version 1.4 [23,24].

#### 2.3.4. Skin biopsy.

The skin biopsy site was determined based on each patient's clinical assessment, and bacilli were identified using the Fite-Faraco stain. As primary neural leprosy patients generally do not show any skin lesions, biopsies were performed near the elbow, as the bacilli have a tropism for areas with lower body temperature [5,6].

#### 2.3.5. Electroneuromyography.

Electroneuromyographic assessments were conducted using the MEB 4200 K (NIHON-KODEN) device at the time of diagnosis. Nerve conduction studies were performed according to established protocols [25]. In the sensory conduction analysis, bilateral evaluations were performed on the median, ulnar, radial, sural, and superficial fibular nerves. The motor conduction study encompassed bilateral assessments of the median, ulnar, common fibular, and tibial nerves. Additionally, targeted techniques were employed to detect focal impairments at compression-prone sites commonly affected in leprosy neuropathy, including the median nerve at the wrist, the ulnar nerve at the elbow, the fibular nerve at the fibular head, and the tibial nerve at the ankle.

Demyelinating impairment was considered when one of the following criteria was present: Prolonged motor distal latency ≥50% above the upper limit of normal values (ULN) (excluding median neuropathy at the wrist from carpal tunnel syndrome); reduction of motor conduction velocity ≥30% below the lower limit of normal values (LLN); prolongation of F-wave latency ≥20% above ULN in two nerves (≥50% if amplitude of distal negative peak CMAP <80% of LLN); motor conduction block defined by a ≥ 50% reduction of the proximal relative to distal negative peak compound muscle action potential (CMAP)amplitude and presence of abnormal temporal dispersion (TD) (characterized by one of the three criteria: multiphasic CMAP; CMAP with duration more than 8 ms and proximal CMAP lasting >20% of the proximal CMAP) [25].

All cases were evaluated according to clinical and neurophysiological criteria according to the European Academy of Neurology/Peripheral Nerve Society (EAN/PNS) guideline on diagnosis and treatment of chronic inflammatory demyelinating polyradiculoneuropathy (CIDP), to discuss the possibility of differential diagnosis with leprosy [26].

#### 2.3.6. Statistical analysis.

Continuous variables were compared using the Mann-Whitney U test (due to non-normal distribution), reporting effect sizes as Hodges–Lehmann median differences with 95% confidence intervals (CIs). Categorical variables were evaluated using Fisher’s exact test, with effect sizes reported as Prevalence Ratios (PR) and 95% CIs. Multivariable binary logistic regression models identified independent predictors for conduction block and temporal dispersion. Before logistic regression analysis, multicollinearity was assessed using the Variance Inflation Factor (VIF). Predictor variables with VIF values ≥ 5 were removed from the analysis to ensure model stability. Data management and statistical analyses were performed using the *Python programming* language (version 3.12), using the pandas and numpy libraries for data manipulation, with a significance level set at 5% (p < 0.05).

## 3. Results

### 3.1. Epidemiological and clinical characteristics

494 participants were included in this study. There was a male prevalence (51%; 252/494), with a mean age of 45.7 (± 16.6) years. All patients presented a non-length-dependent, exclusively or predominantly sensory neuropathy (tactile and painful hypoesthesia). Regarding the presence of LR, 32% (158/494) presented T1LR, while 15.6% (77/494) had T2LR. Considering the evaluation of disability grade, 54% (267/494) presented DG0, 23.9% (118/494) DG1, and 22.1% (109/494) DG2 (Table 1).

**Table 1:** Epidemiological, clinical, and laboratory characteristics among leprosy patients. (n = 494).

Epidemiological data	
Male (%)	51 (252/494)
Female (%)	49 (242/494)
Mean age (years)	45.7 (± 16.6)
**Clinical data**	
Nerve thickening per patient	2.5 (± 2.2)
Motor nerve affected per patient	2 (± 2.5)
Sensory nerve affected per patient	3.2 (± 3.3)
**Operational Classification**	
MB (%)	87.4 (432/494)
PB (%)	12.6 (62/494)
**Leprosy clinical form**	
BT (%)	53.8 (266/494)
BB (%)	6.3 (31/494)
BL (%)	7.5 (37/494)
LL (%)	11.3 (56/494)
PNL (%)	21.1 (104/494)
**WHO leprosy-related impairment disability grade**	
0 (%)	54 (267/494)
1 (%)	23.9 (118/494)
2 (%)	22.1 (109/494)
**Leprosy reaction**	
T1LR (%)	32 (158/494)
T2LR (%)	15.6 (77/494)
**Laboratory**	
Positive ELISA IgM Anti-PGL-I (%)	58.1 (287/494)
Mean ELISA index	1.7 (± 1.7)
Positive Histopathological exam (%)	20 (100/494)
Positive skin bacilloscopy (%)	18.8 (93/494)
Positive skin qPCR (%)	47.4 (234/494)
Positive slit skin smear bacilloscopy (%)	21.3 (105/494)
Positive slit skin smear qPCR (%)	51.6 (255/494)

MB, multibacillary; PB, paucibacillary; BT, borderline-tuberculoid; BB, borderline-borderline; BL, borderline-lepromatous; LL, lepromatous; T1LR, type 1 leprosy reaction; T2LR, type 2 leprosy reaction; qpCR, quantitative polymerase chain reaction

### 3.2. Laboratory analysis

The anti-PGLI IgM ELISA serology was positive in 58.1% (287/494) of the patients, with a mean index of 1.7 (±1.7). The use of the ELISA anti-PGL-I test is justified because it shows high correlation with MB clinical forms and is directly proportional to bacillary load.

The qPCR test of slit skin smear was positive in 51.6% (255/494), and the slit skin smear bacilloscopy in 21.3% (105/494). Regarding the skin biopsy, the qPCR test was also superior to bacilloscopy, being positive in 47.4% (234/494) and 18.8% (93/494), respectively. Skin biopsy showed histopathological abnormalities in 20% (100/494) of the cases (Table 1).

### 3.3. Electroneuromyographic findings

Considering the 494 patients in the study, 3952 nerves were evaluated, with an average of 5.1 (±5.4) nerves affected per patient. There was more significant impairment of sensory nerves (3.2 (± 3.3) nerves/patient) compared to motor nerves (2 (± 2.5) nerves/patient). 6.5% (32/494) presented the pattern of a mononeuropathy, and 93.5% (462/494) an asymmetric multiple mononeuropathy.

116 patients showed a demyelinating pattern defined by standard criteria, and 20.7% (24/116) presented exclusively demyelinating abnormalities without evidence of secondary axonal loss. 81% (94/116) presented conduction block and 95.7% (111/116) temporal dispersion, with the concomitance of both conditions in 76.7% (89/116) of patients. 83.6% (97/116) demonstrated prolongation of distal motor latency, and 99.1% (115/116) reduction in conduction velocity. All these abnormalities were distributed among all evaluated nerves, reinforcing an asymmetric and non-uniform distribution.

### 3.4. Clinical, laboratory, and electroneuromyographic evaluation according to the presence of leprosy reaction

To achieve a better understanding of the relationship between the presence of neurophysiological findings compatible with demyelination and the occurrence of LR, patients were divided into two groups: one composed of individuals presenting LR (43.5%; 215/494) and the other of those patients who did not present LR (56.5%; 279/494) (Table 2).

**Table 2:** Comparative analysis between leprosy patients with and without leprosy reactions.

	With LR (n = 215)	Without LR (n = 279)	Prevalence Ratio (95% CI)	p value
**Clinical data**				
Nerve thickening per patient	3.6 (±2.1)	1.7 (±1.9)		<0.0001**
Motor nerve affected per patient	2.6 (±2.7)	1.5 (±2.2)		<0.0001**
Sensory nerve affected per patient	4.8 (±3.3)	2 (±2.8)		<0.0001**
**Operational Classification**				
MB (%)	94.9 (204/215)	80 (223/279)	2.91(1.70-4.97)	<0.0001**
PB (%)	5.1 (11/215)	20 (56/279)		
**Leprosy clinical form**				
BT (%)	37.7 (81/215)	66.3 (185/279)	0.52 (0.42–0.63)	<0.0001*
BB (%)	7.9 (17/215)	5.0 (14/279)	1.28 (0.89–1.84)	0.1893*
BL (%)	13.5 (29/215)	2.9 (8/279)	1.92 (1.48–2.50)	<0.0001*
LL (%)	24.2 (52/215)	1.4 (4/279)	2.49 (2.14–2.90)	<0.0001*
PNL (%)	16.7 (36/215)	24.4 (68/279)	0.67 (0.49–0.93)	0.0086*
**WHO leprosy-related impairment disability grade**				
0 (%)	35.3 (76/215)	68.5 (191/279)	0.46 (0.38–0.57)	<0.0001*
1 (%)	36.3 (78/215)	14.7 (41/279)	1.79 (1.50–2.15)	<0.0001*
2 (%)	28.4 (61/215)	16.8 (47/279)	1.41 (1.13–1.77)	<0.0001*
**Laboratory**				
Positive ELISA IgM Anti-PGL-I (%)	55.8 (120/215)	59,9 (167/279)		0.2372*
Mean ELISA index	1.9 (±2.1)	1.5 (±1.3)	0.91 (0.75–1.11)	0.1314**
Positive Histopathological exam (%)	34 (73/215)	9.7 (27/279)	2.02 (1.63–2.52)	<0.0001*
Positive skin bacilloscopy (%)	35.3 (76/215)	6.1 (17/279)	2.36 (1.89–2.94)	<0.0001
Positive skin qPCR (%)	60.5 (130/215)	37.3 (104/279)	1.70 (1.41–2.05)	<0.0001
Positive slit skin smear bacilloscopy (%)	39.1 (84/215)	7.5 (21/279)	2.28 (1.85–2.80)	<0.0001
Positive slit skin smear qPCR (%)	56.7 (122/215)	47.7 (133/279)	1.19 (1.00–1.41)	0.0093
**Neurophysiological data**				
Conduction block (%)	27.4 (59/215)	12.2 (34/279)	1.63 (1.23–2.15)	<0.0001
Temporal dispersion (%)	32.6 (70/215)	14.3 (40/279)	1.70 (1.31–2.21)	<0.0001
Conduction block and temporal dispersion (%)	26 (56/215)	11.8 (33/279)	1.64 (1.23–2.19)	<0.0001

MB, multibacillary; PB, paucibacillary; BT, borderline-tuberculoid; BB, borderline-borderline; BL, borderline-lepromatous; LL, lepromatous; T1LR, type 1 leprosy reaction; T2LR, type 2 leprosy reaction; qpCR, quantitative polymerase chain reaction

*Fisher’s Exact Test, **Mann-Whitney U test.

This analysis confirmed a high bacillary load in patients with LR, as observed in the data regarding slit skin smear bacilloscopy, histopathology, and molecular evaluation. Moreover, the group of patients with LR presented more aggressive neural damage, evidenced by the higher average of altered nerves and worse degree of disability.

It is also important to mention that a higher prevalence of demyelinating patterns (conduction block and temporal dispersion) was observed in patients with leprosy reactions (**Table 2**, Suppl. **Table S3**A-**E in** S3 File). The reactional group showed an 83% higher prevalence of conduction block (PR: 1.83; 95% CI: 1.23–2.15; p < 0.0001) and a 70% higher prevalence of temporal dispersion (PR: 1.70; 95% CI: 1.31–2.21; p < 0.0001) compared to the non-reactional group (Table 2).

### 3.5. Clinical, laboratory, and electroneuromyographic evaluation according to the type of LR

Of the 215 patients with LR, 73.5% (158/215) had T1LR, 35.8% (77/215) had T2LR, and 9.3% (20/215) had both reactional states. When comparing the T1LR and T2LR groups, we observed a higher bacillary load in patients with T2LR, as indicated by slit skin smear bacilloscopy, histopathology, and molecular evaluation. Regarding the neurophysiological data, there were no significant differences between the groups (Table 3; Suppl. **Fig S3 in** S1 File). Bacterial load indicators differed significantly between groups. T2LR group exhibited a markedly higher prevalence of positive slit skin smears (PR: 3.19; 95% CI: 2.35–4.31) and skin biopsies (PR: 2.30; 95% CI: 1.68–3.13) compared to T1LR, which appears to corroborate the distinct higher bacillary burden already described for this reactional subtype (Table 3).

**Table 3:** Comparative analysis between leprosy patients with T1LR and T2LR.

	T1LR (n = 158)	T2LR (n = 77)	Prevalence Ratio (95% CI)	p value
				
Positive ELISA IgM Anti-PGL-I (%)	47.5 (75/158)	77.9 (60/77)		<0.0001**
Mean ELISA index	1.5 (±1.7)	3.1 (±2.7)	1.64 (1.33–2.03)	<0.0001**
Positive Histopathological exam (%)	29.7 (47/158)	50.6 (39/77)	1.70 (1.23–2.36)	<0.0001*
Positive skin bacilloscopy (%)	26.6 (42/158)	61 (47/77)	2.30 (1.68–3.13)	<0.0001*
Positive skin qPCR (%)	50.6 (80/158)	87 (67/77)	1.72 (1.44–2.05)	<0.0001*
Positive slit skin smear bacilloscopy (%)	24.1 (38/158)	76.6 (59/77)	3.19 (2.35–4.31)	<0.0001*
Positive slit skin smear qPCR (%)	48.1 (76/158)	75.3 (58/77)	1.57 (1.26–1.94)	<0.0001*
**Neurophysiological data**				
Conduction block (%)	27,2 (43/158)	20.8 (16/77)	0.76 (0.46–1.26)	0.2856*
Temporal dispersion (%)	31 (49/158)	27.3 (21/77)	0.88 (0.57–1.35)	0.5563*
Conduction block and temporal dispersion (%)	25,3 (40/258)	20.8 (16/77)	0.82 (0.49–1.37)	0.4435*

*Fisher’s Exact Test, **Mann-Whitney U test.

### 3.6. Clinical, laboratory, and electroneuromyographic evaluation according to the type of nerve damage

Even though most patients presented with axonal nerve damage (76.5%; 378/494), the demyelinating pattern had a greater clinical impact, with greater neural thickening, more motor and sensory nerves affected, and a worse disability grade.

In laboratory analysis, the demyelinating group had a higher prevalence of positive slit skin smear bacilloscopy and skin bacilloscopy, indicating a higher bacillary load in this group when compared to the group with axonal damage (Table 4, suppl. **Table S2 A**-C in S2 File).

**Table 4:** Comparative analysis between patients with demyelinating neuropathy and axonal neuropathy.

	Demyelinating (n = 116)	Axonal (n = 378)	Prevalence Ratio (95% CI)	p value
**Clinical data**				
Nerve thickening	4 (±1.9)	2 (±2.1)		<0.0001**
Motor nerve alterations	4.7 (±2.6)	1.1 (±1.8)		<0.0001**
Sensory nerve alterations	6.3 (±3.3)	2.2 (±2.7)		<0.0001**
**Operational Classification**				
MB (%)	95.7 (111/116)	83.6 (316/378)	1.14 (1.06–1.24)	0.0001*
PB (%)	4.3 (5/116)	16.4 (62/378)	*Reference*	
**Leprosy clinical form**				
BT (%)	41.4 (48/116)	57.7 (218/378)	0.72 (0.56–0.92)	0.0005*
BB (%)	8.6 (10/116)	5.6 (21/378)	1.55 (0.75–3.20)	0.1525*
BL (%)	8.6 (10/116)	7.1 (27/378)	1.21 (0.60–2.43)	0.1525*
LL (%)	12.1 (14/116)	11.1 (42/378)	1.09 (0.60–1.95)	0.7668*
PNL (%)	29.3 (34/116)	18.5 (70/378)	1.58 (1.11–2.26)	0.0055*
**WHO leprosy-related impairment**				
0 (%)	12.9 (15/116)	65.9 (249/378)	0.20 (0.12–0.32)	<0.0001*
1 (%)	22.4 (26/116)	24.9 (94/378)	0.90 (0.61–1.33)	0.5923*
2 (%)	64.7 (75/116)	9.3 (35/378)	6.98 (4.93–9.89)	<0.0001*
**Leprosy reaction**				
T1LR (%)	45.7 (53/116)	27.8 (105/378)	1.64 (1.26–2.15)	<0.0001*
T2LR (%)	18.1 (21/116)	14.8 (56/378)	1.22 (0.77–1.95)	0.2977*
**Dados laboratoriais**				
Positive ELISA IgM Anti-PGL-I (%)	53.4 (62/116)	59.5 (225/378)		0.1865*
Mean ELISA index	1.9 (±2.2)	1.6 (±1.5)	0.90 (0.73–1.10)	0.3964**
Positive Histopathological exam (%)	25.9 (30/116)	18.5 (70/378)	1.40 (0.95–2.05)	0.0546*
Positive skin bacilloscopy (%)	30.2 (35/116)	15.3 (58/378)	1.97 (1.35–2.86)	<0.0001*
Positive skin qPCR (%)	38.8 (45/116)	47.4 (179/378)	0.82 (0.63–1.06)	0.1052*
Positive slit skin smear bacilloscopy (%)	30.2 (35/116)	18.5 (70/378)	1.63 (1.14–2.33)	0.0025*
Positive slit skin smear qPCR (%)	48.3 (56/116)	52.6 (199/378)	0.92 (0.73–1.15)	0.3539*

MB, multibacillary; PB, paucibacillary; BT, borderline-tuberculoid; BB, borderline-borderline; BL, borderline-lepromatous; LL, lepromatous; T1LR, type 1 leprosy reaction; T2LR, type 2 leprosy reaction; qpCR, quantitative polymerase chain reaction

*Fisher’s Exact Test, **Mann-Whitney U test.

### 3.7. Differential diagnosis with chronic inflammatory demyelinating neuropathy

Among patients with a demyelinating pattern, 93.1% (108/116) fulfill neurophysiological criteria for chronic inflammatory demyelinating polyneuropathy (CIDP). However, no patient meets the clinical criteria for typical CIDP. Despite a chronic and progressive course (over at least 8 weeks), no patient presented with proximal muscle weakness of the upper or lower limbs, nor with absent or reduced tendon reflexes in all limbs.

On the other hand, 46.3% (50/108) of patients with neurophysiological criteria presented clinical criteria for atypical CIDP, notably the multifocal CIDP, also named multifocal acquired demyelinating sensory and motor neuropathy (MADSAM), defined by sensory loss and muscle weakness in a multifocal pattern, usually asymmetric, upper limb predominant, in more than one limb.

The effect sizes for median differences were calculated using the Hodges-Lehmann estimator for reactional versus non-reactional groups, reaction types (T1LR vs. T2LR), and axonal versus demyelinating damage (**Suppl. Tables S2 A–C in** S2 File).

### 3.8. Multivariable logistic regression results

In the multivariable logistic regression analysis, nerve thickening and the WHO disability grade were the most consistent and statistically significant independent predictors for both conduction block and temporal dispersion (p < 0.001). Additionally, male sex was identified as a significant independent risk factor specifically for conduction block (p = 0.008).

The number of thickened nerves demonstrated a positive linear association with neurophysiological impairment. For each additional thickened nerve trunk detected upon palpation, the odds of presenting conduction block increased by approximately 31% (aOR: 1.31; 95% CI: 1.12–1.52), while the odds for temporal dispersion increased by 28% (aOR: 1.28; 95% CI: 1.10–1.47).

Furthermore, patients presenting with higher disability grades showed a threefold increase in the odds of conduction block (aOR: 3.11; 95% CI: 2.18–4.45) and a fourfold increase in the odds of temporal dispersion (aOR: 4.07; 95% CI: 2.85–5.80), suggesting a critical link between cumulative neural damage observed clinically and the neurophysiological evidence of demyelination.

Regarding biological sex, males had significantly higher odds of presenting conduction block than females (aOR: 2.38; 95% CI: 1.25–4.52), although this association was not statistically significant for temporal dispersion (aOR: 1.69; p = 0.087). Other variables, including age, leprosy reactions, bacilloscopy index, ELISA anti-PGL-I, qPCR, and clinical forms, did not show statistical significance in the adjusted models (**Suppl. Tables S3 A–E in** S3 File).

## 4. Discussion

Nerve damage caused by *M. leprae* comprises both demyelinating and axonal neuropathies.[10,11] Numerous studies have already demonstrated that demyelination occurs before the immune response against the bacillus and may be caused solely by the interaction of *M. leprae* or its products with myelinating Schwann cells [29]. From a pathophysiological perspective, early demyelination provides *M. leprae* with a survival advantage and possibly facilitates the progression of infection, as demyelination and subsequent axonal degeneration induce Schwann cell proliferation, which favors invasion, bacterial replication, and subsequent disease progression [27–30].

Mycobacterium leprae exhibits a unique and marked predilection for the peripheral nervous system, driven primarily by its selective affinity for Schwann cells. This tropism is mediated by specific interactions between bacterial surface molecules and components of the Schwann cell basal lamina, particularly laminin-2, which facilitate bacillary adhesion and entry via receptors such as α-dystroglycan. This selective targeting provides a mechanistic basis for the early and often progressive neuropathy characteristic of leprosy. Briefly, the binding of *M. leprae* to myelinated Schwann cell axon units is sufficient to induce demyelination [27,28].

This direct infection induces phenotypic changes in Schwann cells, including dedifferentiation, metabolic dysfunction, and impaired myelin maintenance, resulting in demyelination even in the absence of a clinically evident leprosy reaction. Thus, leprosy neuropathy cannot be attributed exclusively to immune-mediated mechanisms, but also to the direct cytopathic effects of M. leprae on Schwann cells, which justify the occurrence of silent and early neural damage [27,28]. It justifies the presence of demyelination in a significant proportion of cases despite the absence of clinical evidence of LR, as observed in our data.

Although immune responses play a critical role in the clinical manifestations of the disease, identifying non-immune-mediated demyelination induced by *M. leprae* confirms that it is the first, but not the only, mechanism of demyelination. Additionally, it is believed that this contact demyelination destabilizes the neural microenvironment. After that, this triggers a cascade of other cellular responses, recruiting immune cells, particularly the innate immune system cells [27–30].

Our clinical and laboratory data suggest that inflammatory immune reactions play a crucial role in leprosy nerve damage, and that the mere presence of *M. leprae* cannot fully explain the full phenotype observed. The group of patients with leprosy reaction presented a greater number of affected nerves, including motor nerves, justifying a higher degree of disability in this group, which is evidenced by the higher proportion of individuals with GD2. The higher severity of neural impairment suggests a chronic and progressive disease, exacerbated by the numerous inflammatory events that overlap the infection [5,31].

Garbino and Cols evaluated the electroneuromyographic pattern of leprosy patients during LR, defining two distinct pathological myelin findings and two distinct regeneration processes in the reaction groups: In T2LR, the motor conduction block occurred as the main pattern of demyelination, with an acute and focal phenomenon, while on the T1LR the main pattern observed was temporal dispersion, probably due to subacute and chronic segmental demyelination. However, these studies did not compare their findings with cases without clinical evidence of LR [18,32]. Our data also show a higher proportion of LR patients with signs of demyelination (conduction block and/or temporal dispersion), suggesting that this pattern may indicate active disease.

Various acquired conditions can present with features similar to those of leprosy, and in this study, we draw attention to demyelinating inflammatory neuropathies. The differential diagnosis between leprosy and CIDP, particularly its multifocal variants, can be challenging due to overlapping clinical and electrophysiological features. Both conditions may present with asymmetric sensory–motor deficits and segmental demyelination; however, leprosy is distinguished by its predilection for distal peripheral nerves, often with nerve enlargement, sensory loss disproportionate to weakness, and associated skin or autonomic involvement. Furthermore, *M. leprae* can induce demyelination through direct Schwann cell infection, even in the absence of overt inflammation, whereas CIDP represents a primary immune-mediated process. Recognition of these pathophysiological differences, along with careful clinical examination and microbiological or histopathological evaluation, is essential to avoid misdiagnosis and inappropriate immunosuppressive treatment [5,26].

CIDP is primarily a clinical diagnosis characterized by a progressive or relapsing course lasting at least eight weeks, with symmetric or asymmetric proximal and distal weakness, sensory impairment, and reduced or absent deep tendon reflexes. Supportive features include gait disturbance, involvement of both motor and sensory fibers, and functional improvement with immunomodulatory treatment. Importantly, although electroneuromyographic findings of demyelination are essential supportive tools, they should not be interpreted in isolation, as similar patterns may be observed in other neuropathies, including infectious neuropathies. Reliance solely on electrophysiological criteria may therefore lead to misdiagnosis and inappropriate treatment, underscoring the need for careful clinical correlation and exclusion of alternative causes, such as leprosy [26].

Therefore, especially in endemic countries or when there is an epidemiological history of leprosy, when a suspected case of multifocal CIDP is encountered, it is very important to use laboratory tests such as anti-PGL-I serology and molecular evaluation to avoid diagnostic errors. Early diagnosis of suspected leprosy neuropathy cases has always posed a problem due to the long incubation period of the disease, the variable and insidious symptoms, and clinical signs.

Finally, it is essential to emphasize that demyelination is a common pathological feature of peripheral neuropathy, regardless of its infectious or inflammatory etiology. The same features of demyelination observed in our data are also seen in other inflammatory neuropathies, especially in typical and atypical forms of CIDP [26,32].

## 5. Conclusion

Electroneuromyography evaluated in isolation does not allow a correct differential diagnosis between these conditions. Although leprosy is rarely reported in developed countries with low-prevalence settings, where the presence of demyelinating impairment strongly suggests the presence of an inflammatory neuropathy, it should be considered in some situations [33–36]. The diagnosis is challenging in low-incidence countries and often delayed for years. Hence, a clinical evaluation and detailed neurological physical examination are essential, in addition to ENMG and the other laboratory tests used in this study, which enhance diagnostic accuracy.

## Supporting information

S1 File Boxplots of demographic and laboratory variables according to reactional status. (A) Age; (B) Bacilloscopy Index; (C) ELISA Index.
(DOCX)

S2 File Boxplots of demographic and laboratory variables according to axonal and demyelinating neurophysiological patterns. (A) Age distribution (years); (B) Bacilloscopy Index; (C) ELISA Index.
(DOCX)

S3 File Boxplots of demographic and laboratory variables according to leprosy reaction type. (A) Age; (B) Bacilloscopy Index; (C) ELISA Index. **Table S2 A – Effect Sizes: Reactional vs. Non-Reactional. Table S2 B – Effect Sizes: Type 1 vs. Type 2 Reaction. Table S2 C – Effect Sizes: Axonal vs. Demyelinating Damage. Table S3 A.** Assessment of multicollinearity among predictor variables for the Conduction Block outcome. **Table S3 B.** Assessment of multicollinearity among predictor variables for the Temporal dispersion outcome. **Table S3 C. Variance Inflation Factor** (**VIF**) analysis after exclusion of variables with significant multicollinearity (VIF **≥** 5). **Table S3 D.** Multivariable logistic regression analysis of factors associated with Conduction Block. **Table S3 E.** Multivariable logistic regression analysis of factors associated with Temporal Dispersion.
(DOCX)
