# Supplementary material for: Leprosy neuropathy and demyelinating impairment: How should we interpret this neurophysiological pattern?
Source: PLoS One. 2026 Apr 8;21(4):e0343962. doi: 10.1371/journal.pone.0343962 (PMC13061207; doi:10.1371/journal.pone.0343962)
Supplement: S1 File — (A) Age; (B) Bacilloscopy Index; (C) ELISA Index. (DOCX) [file pone.0343962.s001.docx]

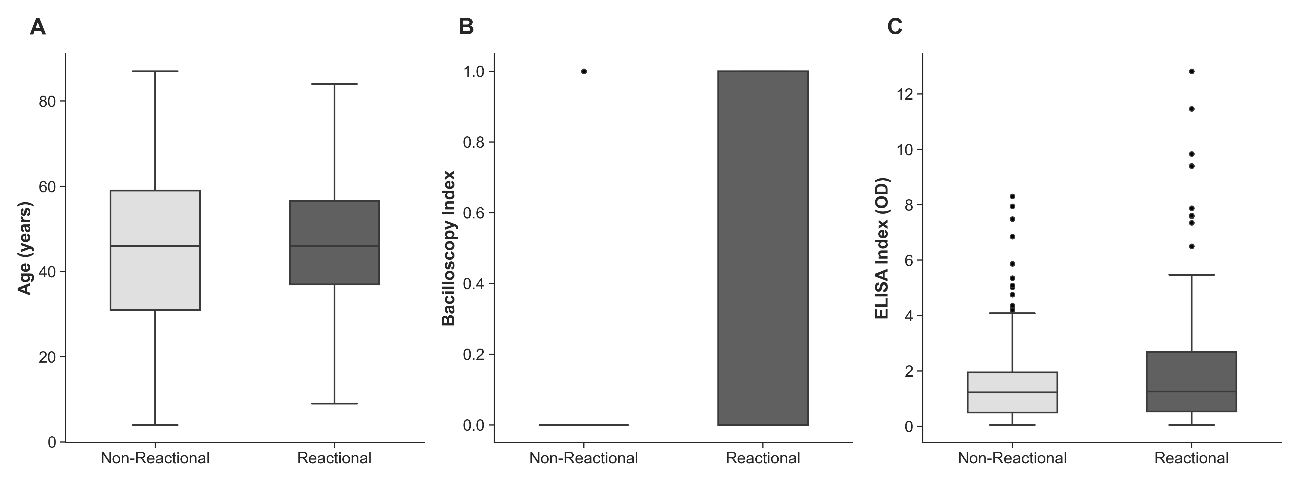


**Fig S1. Boxplots of demographic and laboratory variables according to reactional status.**

(A) Age; (B) Bacilloscopy Index; (C) ELISA Index


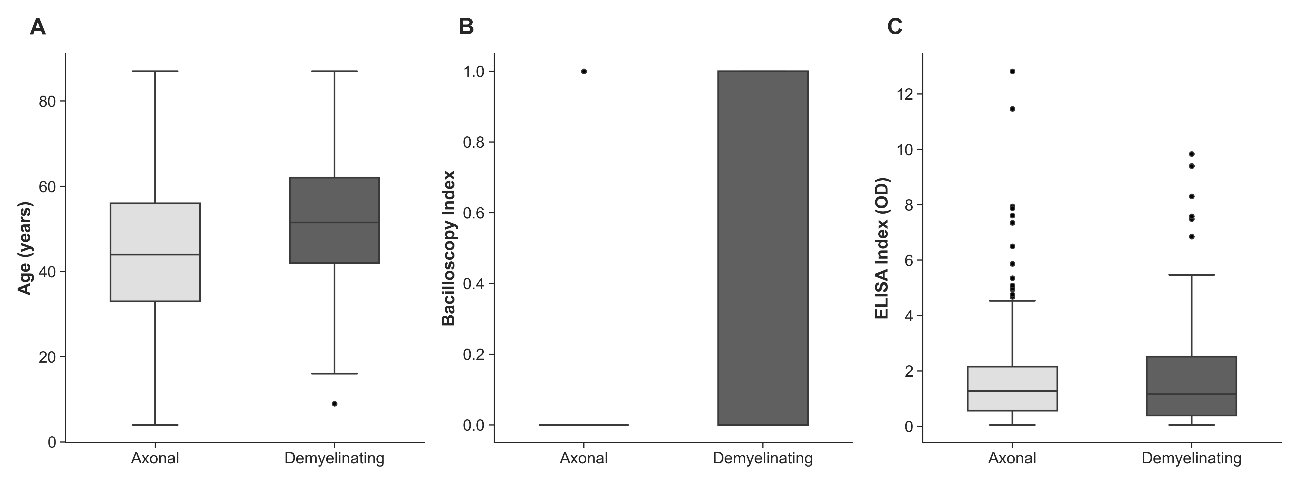


**Fig S2. Boxplots of demographic and laboratory variables according to axonal and demyelinating neurophysiological patterns.**

(A) Age distribution (years); (B) Bacilloscopy Index; (C) ELISA Index


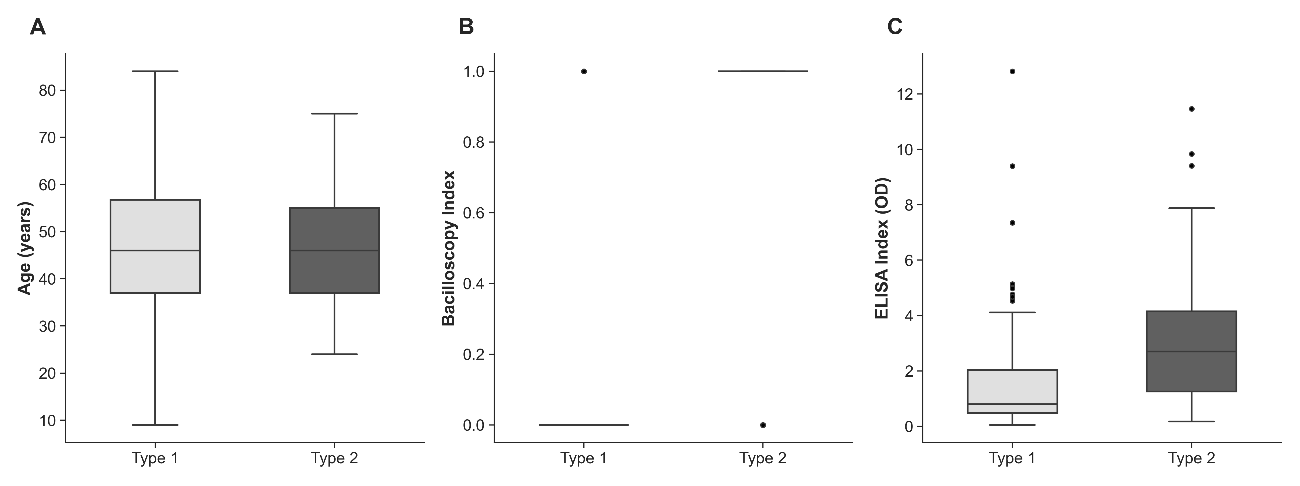


**Fig S3. Boxplots of demographic and laboratory variables according to leprosy reaction type.**

(A) Age; (B) Bacilloscopy Index; (C) ELISA Index.
