# Supplementary material for: Leprosy neuropathy and demyelinating impairment: How should we interpret this neurophysiological pattern?
Source: PLoS One. 2026 Apr 8;21(4):e0343962. doi: 10.1371/journal.pone.0343962 (PMC13061207; doi:10.1371/journal.pone.0343962)
Supplement: S2 File — (A) Age distribution (years); (B) Bacilloscopy Index; (C) ELISA Index. (DOCX) [file pone.0343962.s002.docx]

## **Table S2 A - Effect Sizes: Reactional vs. Non-Reactional**

|  | **Median Difference (Hodges-Lehmann)** | **95% CI** |
| --- | --- | --- |
| Age (years) | 1.00 | -2.00 to 5.00 |
| Nerve Thickening | 2.00 | 2.00 to 2.00 |
| Bacilloscopy Index | 0.00 | 0.00 to 0.00 |
| ELISA Index | 0.12 | -0.04 to 0.29 |

## **Table S2 B - Effect Sizes: Type 1 vs. Type 2 Reaction**

|  | **Median Difference (Hodges-Lehmann)** | **95% CI** |
| --- | --- | --- |
| Age (years) | 1.00 | -3.00 to 5.00 |
| Nerve Thickening | 0.00 | -1.00 to 0.00 |
| Bacilloscopy Index | -1.00 | -1.00 to -1.00 |
| ELISA Index | -1.14 | -1.72 to -0.70 |

## **Table S2 C - Effect Sizes: Axonal vs. Demyelinating Damage**

|  | **Median Difference (Hodges-Lehmann)** | **95% CI** |
| --- | --- | --- |
| Age (years) | -8.00 | -12.00 to -5.00 |
| Nerve Thickening | -2.00 | -3.00 to -2.00 |
| Bacilloscopy Index | 0.00 | 0.00 to 0.00 |
| ELISA Index | 0.08 | -0.11 to 0.26 |
