# Supplementary material for: Leprosy neuropathy and demyelinating impairment: How should we interpret this neurophysiological pattern?
Source: PLoS One. 2026 Apr 8;21(4):e0343962. doi: 10.1371/journal.pone.0343962 (PMC13061207; doi:10.1371/journal.pone.0343962)
Supplement: S3 File — (A) Age; (B) Bacilloscopy Index; (C) ELISA Index. Table S2 A – Effect Sizes: Reactional vs. Non-Reactional. Table S2 B – Effect Sizes: Type 1 vs. Type 2 Reaction. Table S2 C – Effect Sizes: Axonal vs. Demyelinating Damage. Table S3 A. Assessment of multicollinearity among predictor variables for the Conduction Block outcome. Table S3 B. Assessment of multicollinearity among predictor variables for the Temporal dispersion outcome. Table S3 C. Variance Inflation Factor (VIF) analysis after exclusion of variables with significant multicollinearity (VIF ≥ 5). Table S3 D. Multivariable logistic regression analysis of factors associated with Conduction Block. Table S3 E. Multivariable logistic regression analysis of factors associated with Temporal Dispersion. (DOCX) [file pone.0343962.s003.docx]

## **Table S3 A. Assessment of multicollinearity among predictor variables for the Conduction Block outcome**

| **Variável Independente** | **VIF** |
| --- | --- |
| Age | 1.18 |
| Sex | 1.23 |
| Elisa anti-PGL-1 | 1.21 |
| Nerve thickening | 1.73 |
| Bacilloscopy | inf |
| qPCR Biopsy | inf |
| Multibacillary | 1.23 |
| Form BB | inf |
| Form BL | inf |
| Form BT | inf |
| Form LL | inf |
| Form PNL | inf |
| Slit skin smear qPCR | 1.19 |
| Skin qPCR | inf |
| Skin bacilloscopy | 2.52 |
| Skin biopsy | 2.07 |
| T1LR | 1.22 |
| T2LR | 3.19 |
| WHO disability grade | 1.40 |

**Notes**: BT, borderline-tuberculoid; BB, borderline-borderline; BL, borderline-lepromatous; LL, lepromatous T1LR – type 1 leprosy reaction, T2LR – type 2 leprosy reaction, PNL Primary neural leprosy, VIF - Variance Inflation Factor

## **Table S3 B. Assessment of multicollinearity among predictor variables for the Temporal dispersion outcome**

| **Variável Independente** | **VIF** |
| --- | --- |
| Age | 1.18 |
| Sex | 1.23 |
| Elisa anti-PGL-1 | 1.21 |
| Nerve thickening | 1.73 |
| bacilloscopy | inf |
| qPCR Biopsy | inf |
| Multibacillary | 1.23 |
| Form BB | inf |
| Form BL | inf |
| Form BT | inf |
| Form LL | inf |
| Form PNL | inf |
| Slit skin smear qPCR | 1.19 |
| Skin qPCR | inf |
| Slit skin smear bacilloscopy | inf |
| Skin bacilloscopy | 2.52 |
| Skin biopsy | 2.07 |
| T1LR | 1.22 |
| T2LR | 3.19 |
| WHO disability grade | 1.40 |

**Notes**: BT, borderline-tuberculoid; BB, borderline-borderline; BL, borderline-lepromatous; LL, lepromatous T1LR – type 1 leprosy reaction, T2LR – type 2 leprosy reaction, PNL Primary neural leprosy, VIF - Variance Inflation Factor

**Table S3 C. Variance Inflation Factor (VIF) analysis after exclusion of variables with significant multicollinearity (VIF ≥ 5)**

| **Variável** | **VIF** |
| --- | --- |
| Age | 1.14 |
| Sex | 1.20 |
| Nerve thickening | 1.62 |
| WHO disability grade | 1.37 |
| T1LR | 1.17 |
| T2LR | 3.14 |
| Skin Bacilloscopy | 1.91 |
| qPCR Biopsy | 1.25 |
| form BB | 1.20 |
| form BL | 1.67 |
| form LL | 3.08 |
| form PNL | 1.22 |

**Notes**: BT, borderline-tuberculoid; BB, borderline-borderline; BL, borderline-lepromatous; LL, lepromatous T1LR – type 1 leprosy reaction, T2LR – type 2 leprosy reaction, PNL Primary neural leprosy, VIF - Variance Inflation Factor

## **Table S3 D. Multivariable logistic regression analysis of factors associated with Conduction Block**

| **Predictor Variable** | **Adjusted OR** | **95% CI** | **P-value** |
| --- | --- | --- | --- |
| Age | 1.01 | 1.00 – 1.03 | 0.151 |
| Sex (Male) | 2.38 | 1.25 – 4.52 | 0.008 |
| Nerve thickening | 1.31 | 1.12 – 1.52 | < 0.001 |
| WHO disability grade | 3.11 | 2.18 – 4.45 | < 0.001 |
| T1LR | 1.23 | 0.67 – 2.23 | 0.505 |
| T2LR | 1.07 | 0.32 – 3.59 | 0.909 |
| Bacilloscopy | 1.62 | 0.69 – 3.81 | 0.272 |
| Elisa anti-PGL-1 | 1.05 | 0.58 – 1.91 | 0.868 |
| qPCR biopsy | 1.02 | 0.54 – 1.92 | 0.947 |
| Form BB | 1.52 | 0.49 – 4.71 | 0.467 |
| Form BL | 0.46 | 0.13 – 1.61 | 0.224 |
| Form LL | 0.39 | 0.10 – 1.54 | 0.177 |
| Form PNL | 1.64 | 0.80 – 3.34 | 0.176 |

**Notes**: BT, borderline-tuberculoid; BB, borderline-borderline; BL, borderline-lepromatous; LL, lepromatous, PNL Primary neural leprosy, T1LR – type 1 leprosy reaction, T2LR – type 2 leprosy reaction, OR = Odds Ratio; CI = Confidence Interval. Reference category for clinical form is Borderline-Tuberculoid (BT) and female sex

.

## **Table S3 E. Multivariable logistic regression analysis of factors associated with Temporal Dispersion**

| **Predictor Variable** | **Adjusted OR** | **95% CI** | **P-value** |
| --- | --- | --- | --- |
| Age | 1.01 | 0.99 – 1.03 | 0.191 |
| Sex (Male) | 1.69 | 0.93 – 3.09 | 0.087 |
| Nerve thickening | 1.28 | 1.10 – 1.47 | < 0.001 |
| WHO disability grade | 4.07 | 2.85 – 5.80 | < 0.001 |
| T1LR | 1.18 | 0.66 – 2.11 | 0.573 |
| T2LR | 1.01 | 0.31 – 3.28 | 0.989 |
| Bacilloscopy | 2.24 | 0.96 – 5.20 | 0.061 |
| Elisa anti-PGL-1 | 1.25 | 0.70 – 2.26 | 0.449 |
| qPCR biopsy | 0.82 | 0.44 – 1.52 | 0.528 |
| Form BB | 1.20 | 0.39 – 3.70 | 0.754 |
| Form BL | 0.47 | 0.14 – 1.62 | 0.235 |
| Form LL | 0.44 | 0.12 – 1.68 | 0.229 |
| Form PNL | 1.41 | 0.70 – 2.85 | 0.342 |

**Notes**: BT, borderline-tuberculoid; BB, borderline-borderline; BL, borderline-lepromatous; LL, lepromatous, PNL Primary neural leprosy, T1LR – type 1 leprosy reaction, T2LR – type 2 leprosy reaction, OR = Odds Ratio; CI = Confidence Interval. Reference category for clinical form is Borderline-Tuberculoid (BT) and female sex
